# Risk factors for large-for-gestational age infants in pregnant women with type 1 diabetes

**DOI:** 10.1186/s12884-016-0958-0

**Published:** 2016-07-15

**Authors:** Astrid Morrens, Johan Verhaeghe, Christine Vanhole, Roland Devlieger, Chantal Mathieu, Katrien Benhalima

**Affiliations:** Department of Endocrinology, UZ Gasthuisberg, KU Leuven, Herestraat 49, Leuven, 3000 Belgium; Department of Obstetrics & Gynecology, UZ Gasthuisberg, KU Leuven, Herestraat 49, Leuven, 3000 Belgium; Department of Pediatrics, UZ Gasthuisberg, KU Leuven, Herestraat 49, Leuven, 3000 Belgium

**Keywords:** Type 1 diabetes, Pregnancy, Weight gain, Large-for-gestational age infants

## Abstract

**Background:**

The rate of neonatal overweight remains generally high in type 1 diabetes (T1DM). Since glycemic control has improved over time other contributors need to be identified. Our aim is to evaluate the risk factors for large-for-gestational age infants (LGA) in women with T1DM and to evaluate whether the rate of LGA decreased over time.

**Methods:**

Retrospective analysis of the medical files of pregnant women with T1DM attending our university hospital form 01-01-1992 till 31-07-2014. The generalized mixed model was used to adjust for several pregnancies over time in the same women. A multivariable model was used to evaluate independent risk factors for LGA.

**Results:**

Over a 22-year period, 259 pregnancies in 180 T1DM women were identified. Mean diabetes duration of women was 13.7 ± 7.1 years, with a mean age of 29.5 ± 5.2 years. Macrosomia (>4Kg) was present in 16.2 % of deliveries, LGA was present in 45.2 % and these numbers did not change over time (resp. *p* = 0.19 and *p* = 0.70). Over time, significant more women were overweight (23.3 % vs. 39.3 %, *p* = 0.009) and more women had excessive weight gain during pregnancy (21.3 % vs. 37.7 %, *p* = 0.019). Compared to women with a non-LGA baby, women with a LGA baby had a higher weight at delivery (84.1 ± 11.1 vs. 80.4 ± 10.8, *p* = 0.016), had more often excessive weight gain (45.3 % vs. 25.2 %, *p* = 0.003) and had less strict glycaemic control in the first and third trimester [HbA1c of resp. 49 ± 10 mmol/mol (6.7 % ±0.9) vs. 47 ± 8 mmol/mol (6.5 % ±0.8), *p* = 0.01 and 44 ± 5 mmol/mol (6.2 % ±0.5) vs. 42 ± 6 mmol/mol (6.0 % ±0.6), *p* = 0.01]. In the forward multivariable analysis, excessive weight gain [OR 1.95 (1.08–3.53), *p* = 0.027], HbA1c level in early [OR 1.43 (1.05–1.95), *p* = 0.023] and late pregnancy [OR 1.70 (1.07–2.71), *p* = 0.026] remained independent predictors for LGA.

**Conclusions:**

LGA remains a frequent complication in T1DM. Excessive weight gain and HbA1c in early and late pregnancy are important risk factors for LGA in our population. These findings highlight the importance of strict maternal glycemic control and simultaneous striving to appropriate gestational weight gain to minimize the risk of fetal overgrowth in T1DM pregnancies.

## Background

Due to improved medical care, outcomes in pregnant women with type 1 diabetes (T1DM) have improved substantially over the past decades [1–3]. Nevertheless, the prevalence of macrosomia continues to increase [4]. Macrosomia can cause major problems during labor and is associated with increased neonatal morbidity as well as long-term increased risk of developing overweight and type 2 diabetes (T2DM) [5]. Neonatal overweight in T1DM is mainly caused by placental transfer of maternal glucose but since glycemic control has improved over time other contributors need to be identified [6]. In healthy pregnant women obesity and excessive weight gain in pregnancy predispose to macrosomia [7, 8]. Most studies on this topic in women with diabetes include T2DM patients or women with gestational diabetes (GDM) and confirm the strong association between obesity, excessive weight gain in pregnancy and fetal overgrowth [9, 10]. On women with T1DM the evidence is far more scarce. A large Swedish cohort study in T1DM patients showed a positive relation between high pre-pregnancy body mass index (BMI) and the risk of fetal overgrowth [11]. Recently an observational study identified excessive weight gain during pregnancy as an independent risk factor for fetal overgrowth in T1DM patients [12]. The aim of this study was therefore to evaluate the risk factors for large-for-gestational age infants (LGA) in our T1DM population and to evaluate whether the rate of LGA decreased over time. Our hypothesis was that the increasing prevalence of maternal overweight and excessive gestational weight gain, independently of maternal glycemic control, might contribute to the persistent high prevalence of LGA in women with T1DM.

## Methods

Analysis of the electronic medical files over a 22 year period from 01-01-1992 till 31-07-2014 of all pregnant women with T1DM attending the University Hospital UZ Leuven. The study was approved by the Institutional Review Board of UZ Leuven (ML 10676). Our study adheres to the Helsinki Declaration of 1975. Due to the retrospective nature of the study there was no need for informed consent from the participants as in compliance with the Belgian Law of December 8,1992 on the protection of privacy and the Belgian Law of August 22, 2002 on the rights of the patient.

Since 1999 a structured electronic medical file is used in our center. From 1992 till 2012 data were prospectively collected in the database. From 2012 onwards all data were retrospectively collected. Due to lack of data from women with late referrals, only pregnant T1DM women with antepartum follow-up < 12 weeks at UZ Leuven were included in the study. Other exclusion criteria were women with GDM, T2DM, hereditary diabetes, secondary diabetes and use of a peritoneal insulin infusion pump. Because we aimed to analyze the risk factors for LGA, we only included life births and single births, also excluding women with abortion due to congenital malformations, mors in utero and multiple births.

UZ Leuven Hospital is a tertiary care facility with an average of 2400 deliveries per year and is equipped with a High Risk Obstetrics department. Women with T1DM are intensively followed up during and after pregnancy by a multidisciplinary team with an experienced diabetologist, obstetrician and neonatologist using standardized protocols. Women with T1DM are advised to carefully plan their pregnancy. In our center, women with active pregnancy wish are therefore seen monthly at the multidisciplinary diabetes clinic with an experienced diabetologist, diabetes nurse and dietician. The general policy in our center is that contraception can only be stopped when an HbA1c < 53 mmol/mol (7 %) is achieved during at least two consecutive monthly controls. If feasible, a pre-pregnancy HbA1c < 48 mmol/mol (6.5 %) is aimed for. When planning pregnancy and during pregnancy, women are asked to perform self-monitoring of plasma glucose seven times daily (before and 2 h after each meal and at bedtime.). The glycemic targets used in pregnancy are fasting and premeal 3.3–5.5 mmom/l, 2 h postprandial < 6.7 mmol/l and before bedtime 6.0–8.0 mmol/l. From the second trimester on an HbA1c ≤ 42 mmol/mol (6 %) is aimed for. Once pregnant, women are seen two weekly at the multidisciplinary diabetes clinic. In our center an insulin pump is the preferred treatment to optimize glycemic control in pregnancy, unless excellent control on multiple daily injections (MDI). Most women with T1DM are therefore already treated with an insulin pump before pregnancy. As short acting insulin the insulin analogues Aspart or Lispro are used. For women on MDI, since 2011 insulin Detemir is preferably used over insulin Glargine as the long acting insulin analogue. Metformin is rarely used during pregnancy in T1DM in our center and when used this is discontinued at the end of the first trimester.

After the obstetrical booking visit at 6–8 gestational weeks, pregnant T1DM women come for an antepartum visit every 4 weeks until 20–22 gestational weeks, every 2 weeks until 32 gestational weeks and subsequently weekly or twice weekly until delivery. Routine non-stress tests are performed at all visits from 32 gestational weeks onwards and all patients receive a minimum of three ultrasound evaluations. After an uneventful pregnancy, induction of labor is offered to the large majority of women at 38 gestational weeks. In case of complications, particularly hypertension, women are seen at least twice-weekly at the day-ward or admitted to the antepartum ward; the timing of delivery is individualized in these cases. Indications for caesarean section, apart from routine obstetrical indications (breech, etc.), include previous caesarean section and an unfavorable cervix, fetal weight estimated to be LGA on the basis of the abdominal circumference measurement, and severe maternal disease. Over the 22 year follow up period the same standardized protocols were used, except for changes in policy concerning the peripartum glycemic control and hospitalization at the neonatal intensive care. More specifically, since 1999 a strict glycemic control was aimed for during labor and delivery with preferably continuation of the insulin pump. Since the year 2005, provided that the Apgar scores were satisfactory and gestational maturity was achieved, most neonates stay with their mother. Before 2005 almost all neonates were preventively hospitalized at the neonatology department for a few days.

Outcomes were obtained from review of the electronic database. Maternal characteristics recorded were age, ethnicity, weight, BMI at first prenatal visit, overweight (BMI ≥25 and < 29.9Kg/m^2^), obesity (BMI ≥30 Kg/m^2^), weight gain (difference in weight between first prenatal visit and the delivery) and parity. Excessive weight gain was defined according to the most recent Institute of Medicine (IOM) guidelines [13]. Other maternal data that were recorded are: rates of preconception consultation, intake of folium acid (started before or in the first trimester), need for fertility treatment, smoking during pregnancy, diabetes duration, year of delivery, insulin dose (Unit/kg/24 h) in each pregnancy trimester, type of insulin treatment (insulin pump or MDI), HbA1c before pregnancy, HbA1c in each pregnancy trimester (preferably at week 8, week 21 and week 36). Other data collected are the lipid profiles (mostly non-fasting) up to 1 year before pregnancy or in the first trimester (total cholesterol, triglycerides, HDL cholesterol and LDL cholesterol), the blood pressure at week 8, known diabetic retinopathy, known diabetic nephropathy (24 h microalbuminuria >30 mg) and use of steroids.

The following maternal pregnancy outcomes were recorded: pregnancy induced hypertension (PIH) (blood pressure ≥ 140/90 mmHg), preeclampsia (hypertension + proteinuria or in combination with reduced growth or HELPP-syndrome), preterm delivery (<37 weeks of gestation), caesarean sections (primary caesarean sections and total number combining primary and secondary caesarean sections). The following neonatal pregnancy outcomes were recorded: birth weight, gestational age, sex, macrosomia (birth weight > 4 kg), LGA (birth weight >90 percentile according to validated Flemish growth charts adjusted for sex and parity) [14], small for gestational age (birth weight <10 percentile according to validated Flemish growth charts adjusted for sex and parity) [14], Apgar score (five minutes), admission at the neonatal intensive care unit (NICU), hypoglycemia (defined as need of intravenous glucose) and congenital malformations.

To analyze the evolution of pregnancy outcomes over time, the 22 year period was divided in 4 different time periods. Due to changes in policy over time in our hospital, the following division was made: 1992–1998, 1999–2004 since the stricter peripartum glycemic management, 2005–2009 since most neonates stay with their mother and the last 5 years from 2010–2014.

HbA1c was during the study time period measured by different techniques. From 1992 till March 1996 a homemade method was used based on the Jeppson method (Pharmacia Mono S kolom, cation exchange). From March 1996 till May 2002 the immunoassay method Unimate HbA1c of Roche was used (Cobas Integra 400). From May 2002 till August 2007, the immunoassay method Dimension HA1Cof . Dade Behring was used. From August 2007 till August 2014, the reversed-phase cation-exchange chromatography was used (ADAMS HA-8160, Menarini Diagnostics Benelux). Since 1996 Hba1c is reported in compliance with the National Glycohemoglobin Standardization Program [15].

Lipids were during the whole study period measured by the same biomedical technique with also few changes in the calibration methods that were used. Total cholesterol was measured on Cobas 8000, Roche Diagnostics by enzymatic colorimetric CHOD-PAP method, triglycerides by enzymatic colorimetic GPO-PAP method, HDL cholesterol by dextran sulfate/Mg precipitation followed by enzymatic colorimetic CHOD-PAP reaction, calculated LDL-cholesterol by Friedewald formula (Total cholesterol—HDL cholesterol—Triglycerides / 4).

### Statistical analyses

Continuous variables are presented as mean and standard deviation. Normality was assessed by graphical exploration. Categorical variables are presented as frequencies and percentages.

Logistic regression models were used to analyze the association between several clinical variables and LGA as binary outcome. A random intercept for patient was modeled to account for the fact that several pregnancies in the same women may occur in the data set. First, univariable analyzes were performed and secondly, a multivariable model was constructed using a forward model selection strategy, considering variables that resulted significant from the univariable analyzes.

To evaluate differences between 4 time periods with respect to clinical variables, generalized linear mixed models were used for binary variables, and linear mixed models for continuous variables with time period as a factor. To evaluate the relation between the BMI categories (overweight, obesity) and excessive weight gain generalized linear mixed models were used with BMI category as a factor.

Complete case analyses were performed, *p*-values <0.05 were considered significant and all tests were two-sided. Statistical analyses were performed using SPSS 22.0.

## Results

After exclusion of women with late referral after 12 weeks or with follow up in another center (29), 229 women with pregestational diabetes were identified. We further excluded pregnant women with monogenetic forms of diabetes (5), T2DM (28), neonatal diabetes (1), secondary diabetes due to cystic fibrosis (2), a T1DM patient on a peritoneal insulin pump (1), early pregnancy loss due to abortion because of congenital malformations (*n* = 4), mors in utero (4) and multiple births (4). This leaves a cohort of 259 pregnancies in 180 T1DM women over a 22-year period. There were low rates of diabetic retinopathy (2.7 %), diabetic nephropathy (3.9 %), congenital malformations (2.7 %) and Apgar scores < 7 (1.5 % of baby’s).

Table 1 gives an overview of the general characteristics of the whole cohort and the evolution over time using 4 time categories. Missing data were scarce (<5 %) except for the lipid measurements (*n* = 93) and insulin dose in the third trimester (*n* = 40). The majority of women were Caucasian with exception of 8 North African women. 11.6 % of women received a fertility treatment. Insulin pumps were used in 84.6 % of pregnancies. 90 % of women had a preconception consultation. Despite an active policy of pregnancy planning, in 29 % of pregnancies the glycemic control at time of conception was above the target HbA1c of 53 mmol/mol (7 %). Mean diabetes duration of women was 13.7 ± 7.1 years with a mean age of 29.5 ± 5.2 years, both increased over the years. Over time, significant more women were overweight and more women had excessive weight gain during pregnancy. Over time women had a lower mean LDL cholesterol (125.3 ± 20.7 mg/dL vs. 89.2 ± 25.0 mg/dL, *p* = 0.001) and lower levels of triglycerides (118.3 ± 60.3 mg/dl vs. 76.1 ± 35.4 mg/dl, *p* = 0.03). BMI categories overweight and obesity were both a risk factor for excessive weight gain compared to normal weight women (BMI < 25) with an odds ratio (OR) of 4.04 (CI 2.19–7.46, *p* < 0.001) for overweight women and an OR of 5.07 (CI 2.04–12.61, *p* < 0.001) for obese women.

**Table 1:** The characteristics of the whole cohort of type 1 diabetes and evolution over time

	1992-2014	1992-1998	1999-2004	2005-2009	2010-2014	*P* value
	(*n* = 259)	(*n* = 74)	(*n* = 64)	(*n* = 59)	(*n* = 62)	
Duration of Diabetes	13.7 ± 7.1	11.7 ± 5.8	13.7 ± 7.5	13.3 ± 6.7	15.4 ± 8.9	**0.002**
Maternal age at delivery (years)	29.5 ± 5.2	28.7 ± 3.9	29.7 ± 3.7	29.6 ± 4.2	30.4 ± 4.9	**0.03**
Weight trimester 1 (kg)	69.9 ± 10.8	67.8 ± 10.0	70.0 ± 10.7	71.4 ± 10.6	70.7 ± 11.9	**0.003**
Weight at delivery (kg)	82.1 ± 11.1	78.4 ± 9.6	83.4 ± 11.9	85.3 ± 11.3	82.3 ± 10.7	**0.001**
BMI before pregnancy (kg/m2)	25.3 ± 3.8	24.9 ± 3.7	25.5 ± 3.8	25.3 ± 3.6	25.4 ± 4.2	0.12
Obesity	10.9 % (*n* = 28)	9.6 % (*n* = 7)	14.1 % (*n* = 9)	6.8 % (*n* = 4)	13.1 % (*n* = 8)	0.48
Overweight	33.9 % (*n* = 87)	23.3 % (*n* = 17)	28.1 % (*n* = 18)	47.5 % (*n* = 28)	39.3 % (*n* = 24)	**0.009**
Excessive weight gain	34.4 % (*n* = 91)	21.3 % (*n* = 15)	42.2 % (*n* = 27)	40.4 % (*n* = 25)	37.7 % (*n* = 24)	**0.019**
HbA1c before pregnancy mmol/mol (%)	52 ± 10	51 ± 10	53 ± 12	50 ± 6	53 ± 12	0.24
	(6.9 ± 0.9)	(6.8 ± 0.9)	(7.0 ± 1.1)	(6.7 ± 0.6)	(7.0 ± 1.1)	
HbA1c trimester 1 mmol/mol (%)	49 ± 8	46 ± 8	51 ± 11	48 ± 5	50 ± 10	0.05
	(6.6 ± 0.8)	(6.4 ± 0.8)	(6.8 ± 1.0)	(6.5 ± 0.5)	(6.7 ± 0.9)	
HbA1c trimester 2	41 ± 8	39 ± 7	41 ± 7	42 ± 7	42 ± 8	0.23
mmol/mol (%)	(5.9 ± 0.7)	(5.7 ± 0.7)	(5.9 ± 0.6)	(6.0 ± 0.6)	(6.0 ± 0.7)	
HbA1c trimester 3	43 ± 7	41 ± 8	43 ± 7	44 ± 5	44 ± 7	0.10
mmol/mol (%)	(6.1 ± 0.6)	(5.9 ± 0.7)	(6.1 ± 0.6)	(6.2 ± 0.4)	(6.2 ± 0.6)	

*BMI* Body Mass Index, *P* value in bold indicates a statistically significant difference between the four time periods

Results are given as mean (SD) or % (n); Generalized linear mixed models were used for binary variables, and linear mixed models for continuous variables with time period as a factor

Bold indicates statistically significant associations, *p* <0.05

Table 2 gives an overview of the pregnancy outcomes of the whole cohort. The missing data are scarce (<5 %). Macrosomia was present in 16.2 % of women, LGA was present in 45.2 % and 67.2 % received a cesarean section. These numbers did not change significantly over time. Neonatal hypoglycaemia decreased since 1999 due to a stricter glycaemic management peripartum. The NICU admissions decreased since 2005 due to the change in policy in our center.

**Table 2:** The pregnancy outcomes in the whole cohort of women with type 1 diabetes and evolution over time

Outcome	1992-2014	1992-1998	1999-2004	2005-2009	2010-2014	*P* value
	(*n* = 259)	(*n* = 74)	(*n* = 64)	(*n* = 59)	(*n* = 62)	
WOMEN						
Pregnancy induced hypertension	12.7 % (*n* = 33)	16.2 % (*n* = 12)	21.9 % (*n* = 14)	5.1 % (*n* = 3)	6.5 % (*n* = 4)	**0.033**
Pre-eclampsia	8.1 % (*n* = 21)	8.1 % (*n* = 6)	4.7 % (*n* = 3)	8.5 % (*n* = 5)	11.3 % (*n* = 7)	0.55
Preterm delivery	19.7 % (*n* = 51)	21.6 % (*n* = 16)	15.6 % (*n* = 10)	22.0 % (*n* = 13)	19.4 % (*n* = 12)	0.83
Caesarean section	67.2 % (*n* = 174)	68.9 % (*n* = 51)	73.4 % (*n* = 47)	59.3 % (*n* = 35)	66.1 % (*n* = 41)	0.70
Primary Caesarean section	46.3 % (*n* = 120)	52.7 % (*n* = 39)	48.4 % (*n* = 41)	30.5 % (*n* = 18)	51.6 % (*n* = 32)	**0.038**
FETAL						
Male	53.7 % (*n* = 139)	47.3 % (*n* = 35)	59.4 % (*n* = 38)	50.8 % (*n* = 30)	58.1 % (*n* = 36)	0.48
Gestational age at birth (weeks)	37.0 ± 1.4	36.7 ± 1.5	37.1 ± 1.1	37.2 ± 1.5	37.2 ± 1.4	0.35
Birth weight (g)	3458 ± 605	3454 ± 652	3399 ± 649	3462 ± 596	3518 ± 511	0.57
LGA infant	45.2 % (*n* = 117)	50.0 % (*n* = 37)	35.9 % (*n* = 23)	40.7 % (*n* = 24)	53.2 % (*n* = 33)	0.19
Macrosomia	16.2 % (*n* = 42)	17.6 % (*n* = 13)	15.6 % (*n* = 10)	13.6 % (*n* = 8)	17.7 % (*n* = 11)	0.92
Admissions NICU	66.3 % (*n* = 171)	97.3 % (*n* = 72)	79.7 % (*n* = 51)	40.7 % (*n* = 24)	39.3 % (*n* = 24)	**0.001**
Neonatal hypoglycaemia	29.0 % (*n* = 67)	42.6 % (*n* = 20)	28.6 % (*n* = 18)	25.4 % (*n* = 15)	22.6 % (*n* = 14)	0.18

*NICU* neonatal intensive care unit, *LGA* large-for-gestational age infant

Results are given as mean (SD) or % (n); *P* value in bold indicates a statistically significant difference between the four time periods. Generalized linear mixed models were used for binary variables, and linear mixed models for continuous variables with time period as a factor

The difference in characteristics between LGA and non-LGA pregnancies are summarized in Table 3. In the univariable analysis, compared to women with a non-LGA baby, women with a LGA baby had a significantly higher weight at delivery and had significantly more often excessive weight gain. HbA1c in the first and third trimester was also significantly higher in women with a LGA baby. No significant differences in BMI before pregnancy (25.2 ± 3.7 vs. 25.3 ± 3.8, *p* = 0.80), duration of diabetes (13.7 ± 6.7 years vs. 13.7 ± 7.4 years, *p* = 0.99), age at delivery (29.5 ± 4.1 years vs. 29.6 ± 4.3 years, *p* = 0.92), smoking habits (11.1 % vs. 18.3 %, *p* = 0.28), preconception consultation (90.6 % vs. 89.4 %, *p* = 0.65) and lipid levels (LDL cholesterol 87 ± 28 mg/dl vs 91 ± 28 mg/dl, *p* = 0.70, triglycerides 79 ± 36 mg/dl vs. 85 ± 41, *p* = 0.60) were seen between LGA and non-LGA groups. Using a forward model selection strategy, the following variables that resulted significant from the univariable analyzes were included in the multivariable model: weight at delivery, weight gain during pregnancy, excessive weight gain and HbA1c during the first, second and third trimester of pregnancy. In the forward multivariable analysis, excessive weight gain (*p* = 0.027, OR 1.95, CI 1.08–3.53), HbA1c level in early (*p* = 0.023, OR 1.43, CI 1.05–1.95) and late pregnancy (*p* = 0.026, OR 1.70, CI 1.07–2.71) remained independent predictors for LGA.

**Table 3:** The difference in maternal characteristics between LGA and non-LGA pregnancies

Predictor	LGA	Non LGA	OR	CI	*P* value
	(*n* = 117)	(*n* = 142)			
Weight first visit (kg)	70.9 ± 10.6	69.0 ± 10.9	1.01	0.99–1.04	0.25
Weight at delivery (kg)	84.1 ± 11.1	80.4 ± 10.8	1.03	1.01–1.06	**0.016**
BMI before pregnancy (kg/m2)	25.2 ± 3.7	25.3 ± 3.8	0.99	0.92–1.06	0.80
Obesity^a^	11.1 % (*n* = 13)	10.7 % (*n* = 15)	1.01	0.44–2.32	0.99
Overweight^a^	32.5 % (*n* = 38)	35.0 % (*n* = 49)	1.29	0.76–2.18	0.35
Weight gain during pregnancy (kg)	13.2 ± 4.8	11.4 ± 5.2	1.08	1.02–1.13	**0.008**
Excessive weight gain	45.3 % (*n* = 53)	25.2 % (*n* = 38)	2.22	1.29–3.70	**0.003**
HbA1c before pregnancy mmol/mol (%)	53 ± 10	51 ± 11	1.28	0.95–1.72	0.10
	(7.0 ± 0.9)	(6.8 ± 1.0)			
HbA1c trimester 1 mmol/mol (%)	50 ± 10	48 ± 8	1.49	1.10–2.02	**0.010**
	(6.7 ± 0.9)	(6.5 ± 0.8)			
HbA1c trimester 2 mmol/mol (%)	42 ± 7	40 ± 8	1.61	0.98–2.65	0.06
	(6.0 ± 0.6)	(5.8 ± 0.7)			
HbA1c trimester 3 mmol/mol (%)	44 ± 6	42 ± 7	1.82	1.16–2.86	**0.010**
	(6.2 ± 0.5)	(6.0 ± 0.6)			
HbA1c < 42 mmol/mol (6 %) trimester 1	12.7 % (*n* = 14)	25.4 % (*n* = 35)	0.47	0.26–0.86	**0.015**
HbA1c < 42 mmol/mol (6 %) trimester 2	50.9 % (*n* = 56)	67.4 % (*n* = 93)	0.47	0.27–0.80	**0.005**
HbA1c < 42 mmol/mol (6 %) trimester 3	30.1 % (*n* = 31)	40.9 % (*n* = 52)	0.61	0.37–1.02	0.06

*LGA* large-for-gestational-age infant, *BMI* Body Mass Index

Results are given as mean (SD) or % (n); Bold indicates statistically significant associations, *p* < 0.05

Odds ratio (95 %)

^a^reference category: normal weight with BMI < 25 kg/m2

Logistic regression models were used to analyze the association between several clinical variables and LGA as binary outcome

Based on a receiver operating characteristics (ROC) curve for HbA1c in the first trimester with an area under the curve (AUC) of 0.589, a HbA1c ≥ 40 mmol/mol (5.8 %) had the best sensitivity and specificity combined of resp. 94,5 % and 12,7 % to predict a LGA outcome but with a low positive predictive value of 47.7 %. Based on a ROC curve for HbA1c in the third trimester with an AUC of 0.584, a HbA1c ≥ 36 mmol/mol (5.4 %) had the best sensitivity and specificity combined of resp. 97,1 % and 13,4 % to predict a LGA outcome but with a low positive predictive value of 47.6 %.

## Discussion

We show that LGA remains a frequent complication in T1DM. Our study shows that excessive weight gain and HbA1c in early and late pregnancy are independent risk factors for LGA in our population. Despite a generally good glycemic control, the prevalence of overweight women and excessive weight gain during pregnancy increased over the years in our population. Studies in women with pregestational diabetes or gestational diabetes confirm the strong association between obesity, excessive weight gain in pregnancy and fetal overgrowth [9, 10, 16]. Compared to women with T2DM, women with T1DM have generally a higher diabetes duration, a more variable glycaemic control and therefore often a higher HbA1c during pregnancy [17]. More data are therefore necessary to evaluate whether overweight and excessive weight gain are also independent predictors for LGA in women with T1DM. Recently a Danish observational study in 115 women with T1DM identified excessive weight gain during pregnancy as an independent risk factor for fetal overgrowth in T1DM [12]. Our study in a cohort of 259 pregnancies with T1DM shows a high rate of excessive weight gain during pregnancy and confirms the impact of excessive weight gain on fetal overgrowth, independent of maternal glycemic control in early and late pregnancy in T1DM. In addition, our study shows that overweight and obese women often have the highest prevalence of excessive weight gain. This highlights the need for individualized preconception counseling to limit weight gain in these women [18].

In GDM maternal lipids in the third pregnancy trimester have also been shown to be strong determinants of fetal growth [19]. Our study found no relationship between lipid profile and LGA, possibly due to the high number of missing data on the lipid profile.

Glycemic control in early and late pregnancy are major risk factors for LGA in our study population. The majority of our T1DM population was treated with an insulin pump and the glycemic control in our cohort was generally appropriate. However more data are necessary on the most optimal glycemic targets in each trimester. A large prospective study with >700 women with T1DM showed significant increasing rates of LGA with an HbA1c ≥ 42 mmol/mol (6,0 %) at 26 and 34 weeks gestation [20]. In our study, the highest sensitivity to predict LGA was seen at an HbA1c < 42 mmol/mol (6 %) but with a low positive predictive value. Such a strict glycemic control is often challenging to reach since insulin is associated with an increased risk for hypoglycemia and weight gain [21]. New technologies might help to improve pregnancy outcomes but there is currently insufficient evidence that intermittent use of real-time continuous glucose monitoring in T1DM pregnancies can improve pregnancy outcomes [22].

Overweight and obesity are becoming an pandemic problem. In our study the number of overweight T1DM women almost doubled during the study period. This is twice as high as seen in the background population in Flanders [23]. In contrast, the rate of obesity remained stable through the years, similar with the frequency seen in healthy pregnant women in Flanders [23]. Both GDM and maternal obesity are independently associated with adverse pregnancy outcomes [24]. In a large database of 3457 T1DM pregnancies the incidence of LGA increased with greater BMI category [11]. In our study BMI was not an independent risk factor for LGA but this might be due to the rather small number of obese women in our cohort.

Strengths of the study are the detailed characterization of a large cohort with T1DM over more than 20 years. A multivariable model was used to evaluate independent risk factors for LGA. A limit of the study is the retrospective nature of the analysis. However most data were collected prospectively. In our cohort we included women with diabetic nephropathy and pre-eclampsia, which might have underestimated the LGA rate since these conditions have a restrictive impact on birth weight. However numbers were small and this is therefore unlikely to have an impact on the risk factors for LGA. Whether the results are applicable to other populations with T1DM needs to be further explored. Our population consists of relative healthy T1DM patients with generally a good glycemic control and low prevalence of obesity.

## Conclusions

LGA remains a frequent complication in T1DM. Excessive weight gain and HbA1c in early and late pregnancy are important risk factors for LGA in our population. Our findings highlight the importance of strict maternal glycemic control during pregnancy and simultaneous striving to appropriate gestational weight gain to minimize the risk of fetal overgrowth in T1DM pregnancies.

## Abbreviations

BMI, body mass index; GDM, gestational diabetes; HbA1c, hemoglobin A1c; IOM, institute of medicine; LGA, large-for-gestational age; MDI, multiple daily injections; NICU, neonatal intensive care unit; OR, odds ratio; PIH, pregnancy induced hypertension; T1DM, diabetes mellitus type 1; T2DM, diabetes mellitus type 2
